# Influence of the Heterophasic Structure and Its Characteristics on the DC Electrical Properties of Impact Polypropylene Copolymer

**DOI:** 10.3390/polym17070951

**Published:** 2025-03-31

**Authors:** Xinhao Huang, Jiaming Yang, Xindong Zhao, Xu Yang, Kai Wang, Dianyu Wang, Zhe Fu

**Affiliations:** Key Laboratory of Engineering Dielectrics and Its Application, Harbin University of Science and Technology, Harbin 150080, China

**Keywords:** impact polypropylene copolymer, space charge, phase separation, DC conductivity

## Abstract

Space charge injection in polypropylene (PP) significantly weakens the stability of HVDC cables. Impact polypropylene copolymer (IPC) is often used as insulation material for AC cables, but in the DC field, IPC has the problem of space charge accumulation. This is because there is a multi-phase structure inside the IPC to which ethylene monomer was added in the production process, and the difference in physicochemical properties of each phase is an important reason for the accumulation of space charge inside the material. In this work, the vinyl phases and propenyl phases of two types of IPC were separated. The film samples were prepared and tested at 30 °C and 50 °C for DC electrical conductivity, and at 30 °C, 50 °C, and 80 °C for space charge. The experimental results show that the DC conductivity of vinyl phases is significantly higher than that of propenyl phases in both types of IPC. The degrees of mismatch between the DC conductivity of vinyl phase and that of propenyl phase are different in the two types of IPC, and the mismatch degree of DC conductivity is from several times to hundreds of times. The conductivity of the two vinyl samples is ohmic. The conductivity of the two propenyl phases shows nonlinearity under different electric field intensity, and the mismatch degree of the two phases increases with temperature. Compared to untreated IPC, at all test temperatures, the maximum space charge density of the propenyl samples is much lower, which can be reduced by about 1/3 at 50 °C and by about 50% at 80 °C. The density of heteropolar charge produced by impurity ionization in the samples and the depth of electrode injection both decreased. At each temperature, the distortion rate of the electric field in propenyl samples is lower than that in IPC, the distortion rate can be reduced by more than 15%, and the distortion rate can be reduced by nearly half at 80 °C. The charge dissipation characteristic of propenyl samples during depolarization is also optimized compared with IPC samples, the time required for charge dissipation to reach stability is shortened, and the residual charge density in the sample is reduced at the end of depolarization. In addition, the relevance between the variation of DC conductivity of phases and space charge characteristics was discussed according to SCLC (space charge limited current) theory. This work provides a feasible reference for the manufacture of high-reliability polypropylene-based cable material with excellent insulation performance.

## 1. Introduction

The space charge injection under high temperature and high electric field conditions is the main obstacle for the development of high-voltage direct current (HVDC) cables, which would lead to local electric field distort, and in severe cases, partial discharge would produce an insulation break down [1,2,3,4,5,6].

The Unipol polypropylene process is a major worldwide manufacturing technique for PP production today which can produce various kinds of PP, including homopolymer polypropylene (H-PP), polypropylene random (PP-R) copolymer and impact polypropylene copolymer (IPC). It is different from the polyethylene (PE) production process, which directly polymerizes ethylene under ultra-high pressure. PP is usually polymerized from propylene monomers under the Ziegler–Natta catalyst system [7]. In the production process of IPC, the PP molecular chain is initiated by the catalyst (mostly TiCl4), an α-olefin such as ethylene is added into the polymerization reaction, and the simple PP molecular chain grows under the condition of cocatalyst (mostly triethylaluminum) and finally forms the long-chain structure. Therefore, the multi-phase structure occurs in IPC [8,9].

IPC is a microscale mixture composed of homopolymer polypropylene (H-PP), ethylene-propylene random copolymer (EPR), and ethylene-propylene block copolymer (EbP) with different length crystallizable sequences. For IPC, H-PP is the main component, acting as the substrate of the material, while EPR, EbP is the dispersed phase in the material. In order to facilitate the study, the matrix of material is called the propenyl phase, and the EPR and EbP as the dispersed phase are collectively called the vinyl phase [10]. The difference of electrical properties between the vinyl phase and the propenyl phase will affect the overall electrical properties of IPC. According to the current continuity equation,

(1) $$\nabla \cdot J+\frac{\partial \rho }{\partial t}=0$$

where ***J*** is the volume current density, and *ρ* is the volume charge density. Different direct current (DC) conductivity of each phase will produce different conductivity currents and different current density, resulting in the accumulation of space charge at the interface.

The vinyl phase is an amorphous phase that cannot be crystallized into a deep trap. Zhao [11,12] et al. characterized charge storage behavior by electrostatic force microscopy and suggested that space charge can be suppressed by a charged lattice formed by a uniform, dense and fixed distribution of charge trapped by a deep trap. PP with high vinyl phase content can be considered to have a lower trap density, and the ability to capture charge carriers if the material is weaker. At the same time, the existence of more interface structures in PP will introduce more interface defects, which will lead to uneven electric field distribution, local high field intensity, and increased conductance and loss. The defects are more likely to accumulate charge, reducing reliability of the insulation.

This article makes an effort to detach the vinyl phase and the propenyl phase of IPC by etching to investigate the effect of different DC conductivity on the space charge properties of multi-phase structures. The etching solutions were prepared by n-heptane. Then, the etching treatment was carried out by the magnetic stirring method, and the treatment parameters, including the grain diameter of PP powder, etching temperature and etching time, were optimized through the DC conductivity test of etched samples. The DC conductivity of the vinyl phase and the propenyl phase were measured and calculated, respectively. In order to characterize the effect of the etching treatment, a scanning electron microscope (SEM) was used to measure the vinyl phase residue in the etched propenyl phase sample. Finally, the space charge characteristics of the propenyl phases after removing the vinyl phases were tested. This study is of great significance for the development of superior performance PP insulation material for HVDC.

## 2. Experimental

### 2.1. Materials

PP used in this work was from two types of IPC from Sinopec Co., Ltd., Beijing, China, code-named B and L, respectively. The density of B is 0.89 to 0.91 g/cm^3^, and the volume resistivity at room temperature is about 0.5 to 1.0 × 10^15^ Ωm. The density of L is 0.89 to 0.91 g/cm^3^ as well, and the volume resistivity at room temperature is about 0.4 to 1.3 × 10^15^ Ωm.

The etching solution was n-heptane [CH_3_(CH_2_)_5_CH_3_] with a purity of ≥99%, purchased from Tianjin Fuyu Fine Chemical Co., Ltd., Tianjin, China.

### 2.2. Sample Preparation

**Figure 1:**
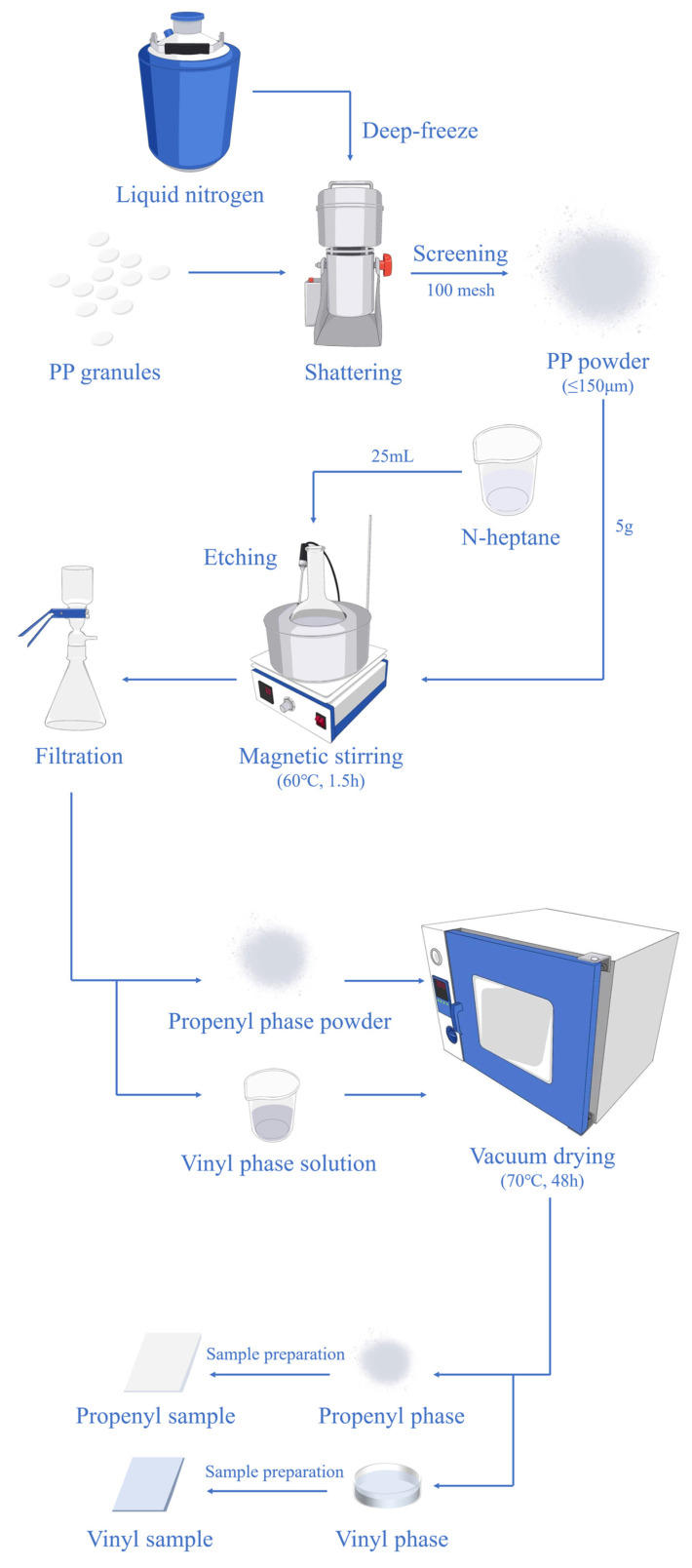
Schematic of separation process of vinyl phase and propenyl phase in PP.

The samples were prepared with PP powder (particle size: 100 mesh, grain diameter: ≤150 μm). The PP powder was obtained by shattering the PP granules. The deep-freezing and shattering process and the etching separation process are shown in Figure 1. First, the PP granules were put in the multifunctional pulverizer (volume: 500 mL). Then, the pulverizer was filled with enough liquid nitrogen to deep-freeze the PP granules to make them brittle enough to crush into powder. After each shattering, a 100-mesh screen was used to screen the powder with a grain diameter less than 150 μm; then, the remaining powder was put back into the multifunctional pulverizer and liquid nitrogen was added for freezing and the next shattering. The steps above were repeated until a sufficient amount of PP powder with a particle size meeting the requirements was harvested.

After the preparation of PP powder, 5 g of the PP powder and 25 mL of the etching solution were successively put into a conical flask, which was fully mixed in a magnetic stirrer at 1.5 h. The etching solution was n-heptane. Then, the etched powder was filtered with a filter screen, and the filtration process was repeated three times to ensure that the propenyl phase and the vinyl phase could be completely detached into two separate parts. It should be noted that the contents that can be dissolved and separated by n-heptane are collectively called vinyl phase in this work, not any one component in particular. These components play a role in improving the mechanical properties of PP materials, the difference of electrical properties between these components and propenyl phase will affect the electrical properties of PP material. Finally, to remove the residual n-heptane, the filtered propenyl phase powder and the vinyl phase solution were dried in a vacuum oven at 70 °C and −0.1 Mpa for 48.0 h.

The etched powder was hot-pressed in molds of different specifications using a flat vulcanizing machine at 180 °C under pressure increasing step-by-step by 5, 10, and 15 MPa, each stage held for 3 min 20 s. Then, the sample was rapidly cooled with a water cooling machine, also under the conditions of 15 MPa. Finally, the vinyl phase sample and the propenyl phase sample with a thickness of ∼200 μm was obtained for test.

### 2.3. Experimental Setup

**Figure 2:**
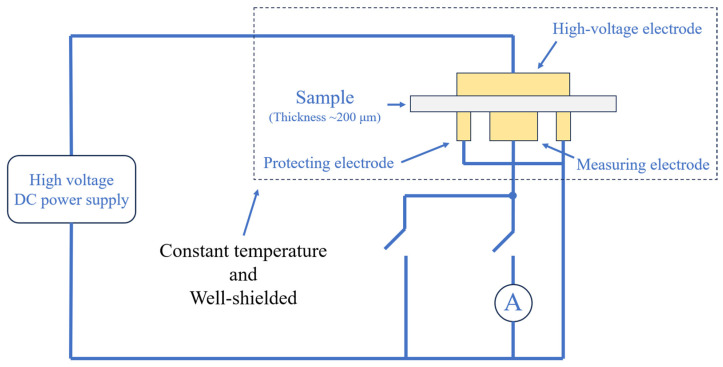
Schematic of the 3-electrode system.

The DC conductivity test was carried out with the three-electrode system at 30 °C and 50 °C. The three-electrode system is a conductance current testing system composed of three well-shielded metal electrodes, high-voltage DC power supply and a piammeter. The three electrodes are a cylindrical high-voltage electrode and a measuring electrode, and a circular protecting electrode. A schematic of the system is shown in Figure 2.

The diameters of the high-voltage electrode and measuring electrode used in this work were 80 mm and 50 mm, respectively, and the outer and inner diameters of the protecting electrode were 74 mm and 54 mm, respectively. The high-voltage DC power supply adopted PS370 produced by SRS, Sunnyvale, CA, USA. The current measurement was measured by EST122 picoammeter, produced by Beijing PLC Co., Ltd., Beijing, China. The test adopted a stepwise step-up mode, each of the samples were tested at an electric field strength of 5, 7.5, 10, 12.5, 15, 17.5, 20, 25, 30 and 35 kV/mm. After the voltage was applied, the current flowing through the sample was recorded within 15 min.

To analyze the composition of the treated sample, the FTIR spectra in the wavenumber range of 4000–400 cm^−1^ were measured with a resolution and scan number of 2 cm^−1^ and 30, respectively, using a Thermo Scientific Nicolet iS20 produced by Beijing SJC Science and Trade Co., Ltd., Beijing, China.

The microstructure of untreated PP and etched propenyl samples was observed by scanning electron microscope (SEM). The samples were frozen in liquid nitrogen for 30 min and embrittered, etched in n-heptane at 60 °C for 15 min, and then ultrasonic cleaned in deionized water for 15 min. The cross-section of the samples was sprayed with gold and observed under an electron microscope. SU8020 SEM produced by Hitachi High-tech Group, Tokyo, Japan, was used.

The space charge test was carried out to verify the effect of interphase conductivity mismatch on space charge characteristics. The pulsed electro-acoustic method (PEA) was used to test the space charge characteristics of the propenyl samples and untreated IPC samples under a 40 kV/mm electric field at 30 °C, 50 °C and 80 °C. The polarization time was 30 min, and the depolarization time was also 30 min.

To simplify the description, the propenyl phases in IPC B and L were numbered BP and LP, respectively. The vinyl phases in IPC B and L were numbered BV and LV, respectively.

## 3. Experimental Results and Discussion

### 3.1. FTIR Spectra

**Figure 3:**
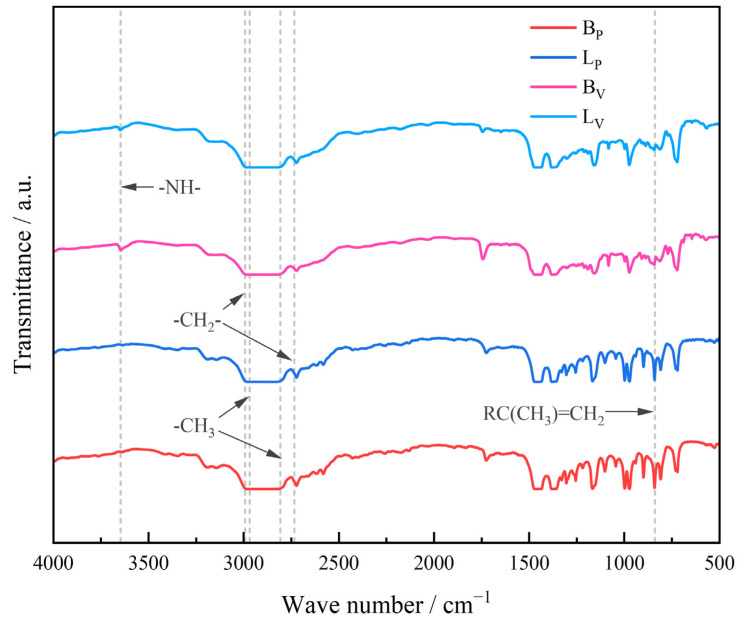
FTIR spectra (transmission mode) of propenyl phase and vinyl phases s in two types of IPC.

The FTIR spectra (transmission mode) of BP, LP, BV and LV are shown in Figure 3. Before testing the samples, an infrared spectrum test was first performed on the air to eliminate background noise, removing the effects of impurities including moisture and others. The typical infrared absorption bands produced by the stretching and vibrations of methyl and methylene are found in all four samples, with absorption peaks at 2953 cm^−1^, 2971 cm^−1^, 2873 cm^−1^, 2845 cm^−1^ and 1375 cm^−1^. These correspond to the asymmetric stretching vibration of -CH_3_, the asymmetric stretching vibration of -CH_2_-, the symmetric stretching vibration of -CH_3_, the symmetric stretching vibration of -CH_2_- and the bending vibration of C-CH_3_. The absorption band is strongest at 720 cm^−1^ illustrating that the methylene sequences connected in a straight chain with lengths greater than -(CH_2_)_7_- are dominant [13,14].

As seen in Figure 3, an absorption peak at 855 cm^−1^ could be found in the infrared spectra of BP, mostly corresponding to the end group RC(CH_3_)=CH_2_. There is no obvious absorption peak at 855 cm^−1^ in the infrared spectra of BV. This phenomenon shows that the vinyl phase in B does not contain double bond structure. The vinyl phase in B may have a similar structure to ethylene propylene binary rubber (EPR) [15,16]. The same difference occurs in the two phases of L.

A series of overlapping absorption bands between 753 and 720 cm^−1^ are produced by a sequence of different lengths of -CH_2_-. This indicates that BV and LV may contain short molecular chain polymers polymerized from ethylene, butene, hexene and other α-olefin [17].

In the process of sample preparation, we found that the self-bonding rate of the vinyl phase samples is relatively low, which could indicate that the vinyl phases are dominated by short-chain structures.

### 3.2. DC Electrical Conductivity

**Figure 4:**
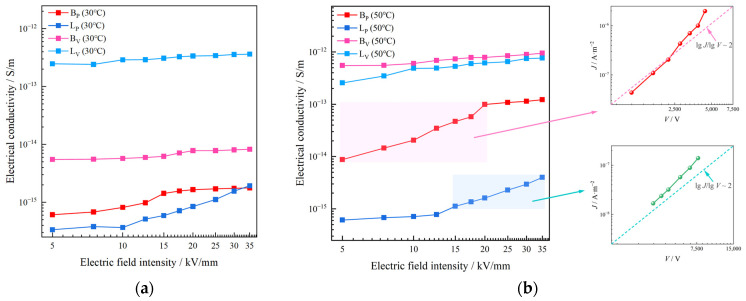
DC conductivity of propenyl phase and vinyl phase in two types of IPC at (**a**) 30 °C, (**b**) 50 °C.

Since the melting temperature of the vinyl phases in two types of IPC is between 50 °C and 70 °C, the DC electrical conductivity of BP, LP, BV and LV at 30 °C and 50 °C was tested in this work, as shown in Figure 4a,b. In this section, the data directly measured by the experiment were the volume conductance current. The volume conductivity and current density under different electric field intensity were calculated by the following formulae:

(2) $$J=\frac{I}{A}=\frac{I}{19.625\times 10^{-4}}$$

Equation (2) is the formula of the volume current density, where I is the volume conductance current, and A is the area of the contact surface between the samples and the measuring electrode used in this work; the value was 19.625 × 10^−4^ m^−2^.

(3) $$\gamma =\frac{J}{E}$$

Equation (3) is the formula of the volume conductivity, where J is the volume current density, and E is the electric field intensity.

Both BV and LV have a higher conductivity than BP and LP at 30 °C. The conductivity of BV and LV show almost no variation with the increase of the electric field intensity, and is in ohmic region. The conductivity of LP shows a great surge when the electric field intensity enters a relatively high level of ≥12.5 kV/mm, as shown in Figure 4a. The DC conductivity of BV is 4.3 to 8.9 times that of BP, and the conductivity of LV is 187.6 to 736.2 times that of LP with the electric field intensity varying from 5 to 35 kV/mm at 30 °C.

As seen in Figure 4b, the conductivity of BP and BV shows obvious temperature sensitivity. When the test temperature rose from 30 °C to 50 °C, the overall conductivity of BP and BV increased by more than 10 times and more than 100 times, respectively. Similarly to the test at 30 °C, BV still shows a higher conductivity than BP. The conductivity of BP significant rises with respect to electric field intensity at relatively low electric field intensity (≤20 kV/mm). When the electric field intensity increases to a high level (≥20 kV/mm), the conductivity of BP shows a small nonlinearity. The conductivity of BV shows no obvious nonlinearity at all. In general, the DC conductivity of BV is 7.7 to 62.8 times that of BP with the electric field intensity varying from 5 to 35 kV/mm at 50 °C.

Differently from B, LP and LV show no obvious temperature sensitivity. LV has a higher conductivity than LP. Nonlinearities of the conductivity of both LV and LP are small at a low electric field intensity (≤12.5 kV/mm). When the electric field intensity increases to a relatively high level (≥12.5 kV/mm), the conductivity of LP shows a significant nonlinearity. The conductivity of LP increases significantly with the intensity of the electric field, but always maintains a lower level than LV. The conductivity of LV is 191.3 to 631.3 times that of LP with the electric field intensity varying from 5 to 35 kV/mm at 50 °C.

The mismatching of DC conductivity between the two phases in L is more obvious, and the degree of conductivity mismatch between the two phases increases with temperature both in B and L.

The high isotropy of propenyl phase results in the formation of crystals in the interior, and the formation of spherulites introduces more interfacial traps, which increases the probability of carrier being caught during the transport process, thus contributing to the decrease of conductivity [18]. Therefore, the propenyl phases in both B and L have a much lower DC conductivity than the vinyl phases.

It can be noted that the DC conductivity of the propenyl phases in both IPCs have a region that increased rapidly with the electric field intensity. This phenomenon could be explained by the space charge limiting current. The SCLC (space charge limited current) law was proposed by Mott and Gurney in 1941 [19]: When the applied voltage gradually increases, that is, when excessive carriers are injected from the injection electrode into the medium with relatively low carrier mobility, part of the carriers will not flow from the extraction electrode to the external circuit but will reside in the medium, forming space charges and obstructing the injection of subsequent charges. Mott-Gurney’s law is shown as Equation (4) below:

(4) $$J=\frac{9}{8}\varepsilon \;\mu \frac{V^{2}}{L^{3}}$$

where *J* is the current density, *ε* is the dielectric constant, *μ* is the carrier mobility, *V* is the applied voltage, and *L* is the thickness of the medium [20].

According to Mott–Gurney’s law, if the DC conductivity of the propenyl phases was affected by the space charge limited current, log⁡J should be approximately linearly related to log⁡V and have a slope of approximately 2.

As seen in Figure 4b, the log⁡J-log⁡V of BP has a slope of a little more than 2 with the electric field intensity range from 5 to 20 kV/mm, while LP has a similar slope of a little more than 2 with the electric field intensity range from 12.5 to 35 kV/mm. This phenomenon could be illustrated by trap-acting space charge limited current (TL-SCLC): in the TL-SCLC region, part of the charge injected by the electrode exists in the form of conductance current, and the other part is filled in traps and contributes the current component in the way of trapping, so that the current density shows a strong nonlinear variation with the electric field [21,22,23].

In conclusion, we could consider that the nonlinear region of DC conductivity of the propenyl phase is mainly affected by the space charge limiting current.

It can be noted that after the nonlinear region ends, the conductivity of BP enters the ohmic region again. This phenomenon could also be illustrated by space charge limited current. In the case of the effect of space charge, the current through the space charge region is mainly the carrier drift current, and the electric field that determines the drift current is mainly generated by the carrier charge. Under the space charge effect, if the space charge density is ρ, the corresponding drift current density *J* is determined by the space charge (let the electron drift velocity be *v*): *J* = *ρ v*. At lower electric fields, drift velocity also depends on electric field intensity *E* (*v* = *μ E*), and we could obtain Mott–Gurney’s law above. However, when the electric field strength increases to a relatively high level, the drift velocity is independent of the electric field intensity, and the velocity is saturated (*v* = *v_sat_*) in this case, then the drift current is proportional to the voltage as follows:

(5) $$J=2q\;\varepsilon \;v_{sat}\frac{V}{L^{2}}$$

The DC conductivity of the propenyl phase enters the ohmic region again. The theory above could also explain the reason why the current density of the vinyl phase is linear with the electric field intensity throughout the test range. In the non-crystallized vinyl phase, the carrier motion lacks the restriction of collision with the lattice, carrier mobility is extremely high, and maximum velocity could be achieved at relative field intensity. The relation between current density and field intensity follows Equation (5).

### 3.3. Space Charge Characteristics

**Figure 5:**
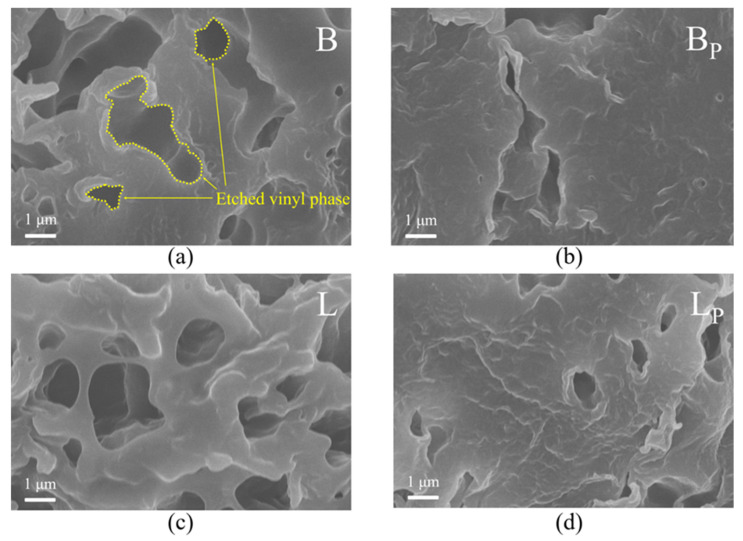
SEM images of (**a**) IPC B, (**b**) propenyl sample of B, (**c**) IPC L, (**d**) propenyl sample of L.

In order to directly show the influence of interface structures on the space charge characteristics of PP materials, the space charge properties of two untreated IPCs, in which much vinyl phase exists in the material, and two propenyl samples with most of the vinyl phase removed, were tested at three different temperatures. In this work, ultrasonic etching was used to remove the vinyl phase in IPC samples and the residual vinyl phase in propenyl samples, and the interface structure could be characterized by the size and density of etched holes, as shown in Figure 5. The microscopic images of the samples after etching were observed by SEM. It should be noted that the etching process mentioned above was just used to characterize the interface structures inside the IPC samples and propenyl samples. The IPCs used for the subsequent space charge test were not etched. None of the samples used for the space charge test were etched after sample preparation, and there were no holes inside the samples.

There are relatively obvious etch holes in the local area of the IPC samples but few in the propenyl samples. According to the etched holes, the vinyl phase in B is mostly irregular long bars with lengths ranging from 2.5 to 10 μm, and the cross section is mostly approximately circular with a diameter of no more than 1 μm. There are no obvious holes in BP, as shown in Figure 5, so it could be considered that almost no vinyl phase exists in BP. The vinyl phase in L is mostly irregular spherical with a diameter of more than 1 μm, and exists in L with a relatively high density. Because of the high content of the vinyl phase, a small amount of vinyl phase still remained in LP after separations, with a diameter of no more than 0.5 μm, and low density. It could be said that the etching separation method used in this article has a good effect in separating the vinyl phase from the propenyl phase in IPC. The density of interface structures in propenyl samples was much lower than that of the IPC samples.

**Figure 6:**
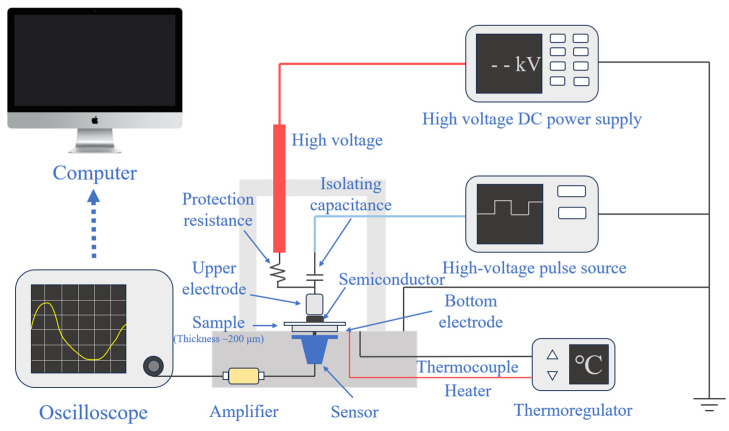
Schematic of the Pulsed Electro-Acoustic (PEA) method.

**Figure 7:**
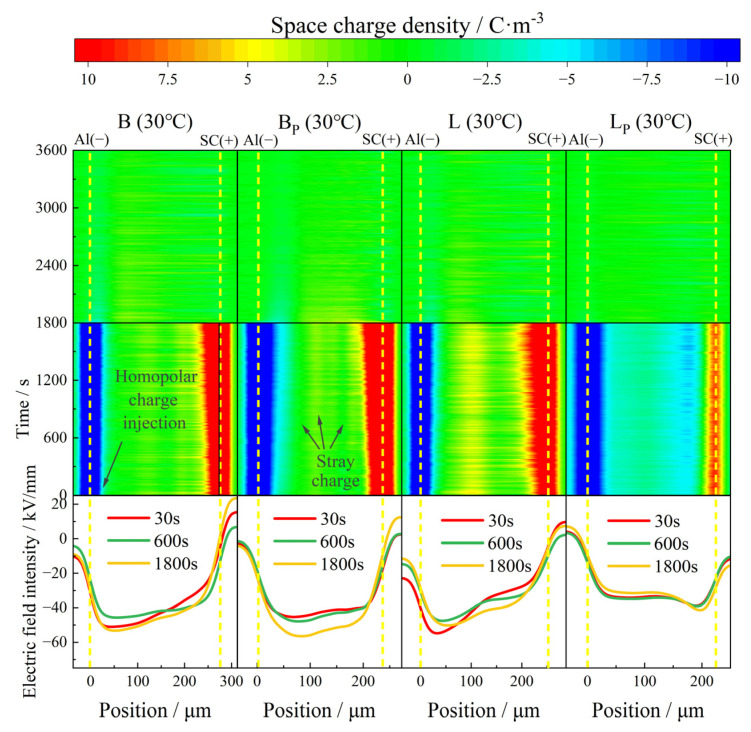
Space charge and electric field distribution of propenyl phases and untreated IPC at 30 °C.

**Figure 8:**
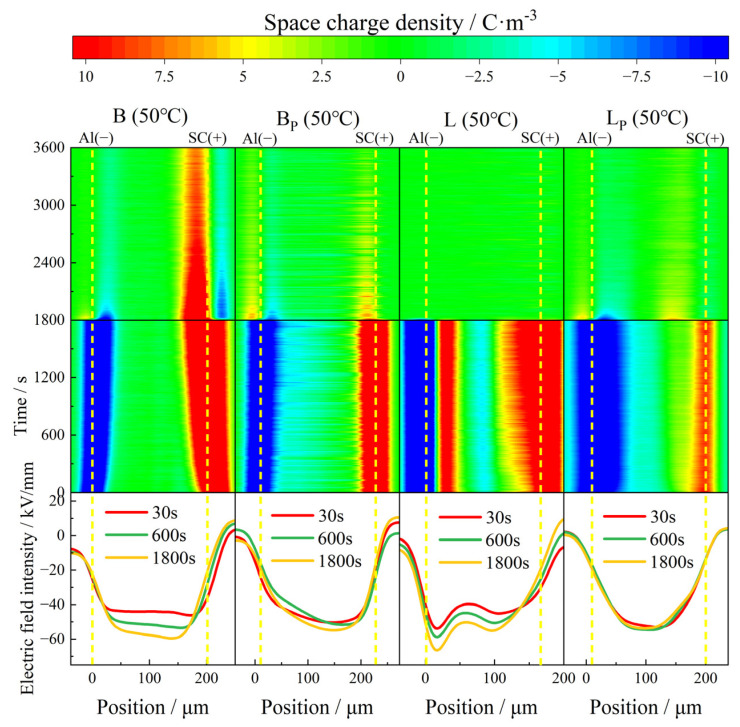
Space charge and electric field distribution of propenyl phases and untreated IPC at 50 °C.

**Figure 9:**
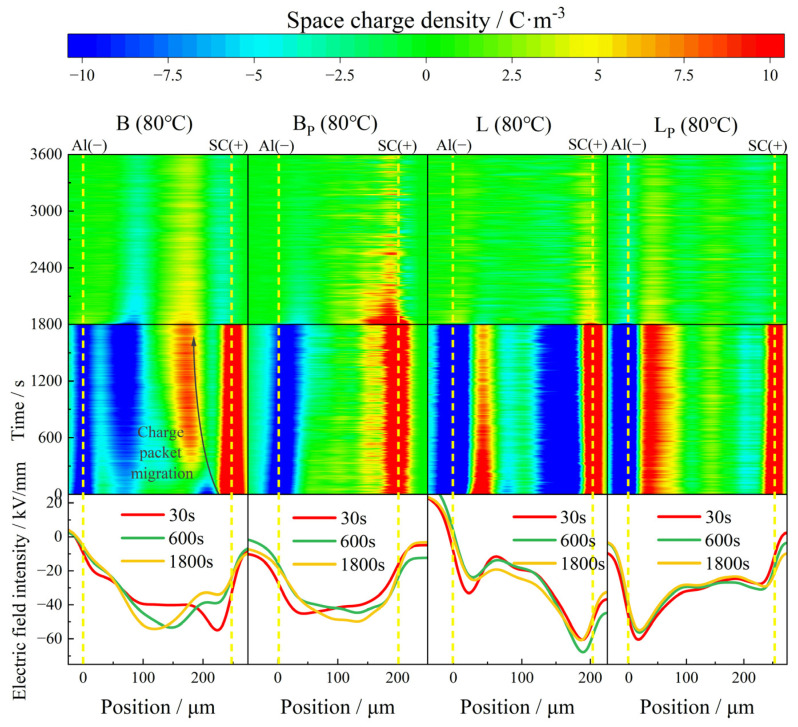
Space charge and electric field distribution of propenyl phases and untreated IPC at 80 °C.

The Pulsed Electro-Acoustic (PEA) method was used to measure space charge characteristics. The basic principle is to add an electric pulse to the electrodes at both ends of the medium. The space charge in the medium and the electrode interface are affected by the pulsed electric field force, and sound pulses are generated accordingly. The space charge distribution information in the medium can be obtained by receiving and measuring these acoustic pulses with piezoelectric electro-acoustic pulse sensors (usually wide-band PVDF piezoelectric film sensors). A temperature-controlled DC electric field type flat plate space charge testing system based on PEA method was used in this work. The electrodes were aluminum electrodes, and the upper electrode and samples were pad with ±525 kV XLPE semi-conductive shielding material produced by the Nordic Chemical Industry to ensure the matching of acoustic impedance. The electric field strength of the pulse source was set at 1 kV/mm and the width was 3 ns. The test principle is shown in Figure 6. The samples were square wafers, 4 to 5 cm long on each side and about 200 μm thick, and space charge characteristics were measured along thickness directions, corresponding to the position axis in Figure 7, Figure 8 and Figure 9.

The accumulated space charge inside the material can be divided into heteropolar charge and homopolar charge. The heteropolar charge mainly comes from the impurity dissociation inside the material, and the homopolar charge mainly comes from the electrode injection. According to the SCLC theory, under the action of a DC electric field, charge transport in the medium is divided into ohmic conductance region and space charge limited current region with the increase of the electric field. The reason for the transformation of this charge transport mechanism is that charge is injected into the material, and the field intensity is the threshold field intensity of the space charge injection. In this section, the test field intensity is 40 kV/mm, which exceeds the threshold field intensity of the space charge injection, so as to better study the influence of interface structures on the space charge characteristics [24,25]. Color-fill diagrams were used to represent the distribution of the space charge with respect to time and position. The color spectrum represents the polarity and density of charge. The density and depth of the injected charge, the stray charge in the interior of the samples and charge packet migration, the deepening of the injection with the extension of the polarization, and the dissipation of the space charge with the depolarization were comprehensively considered to evaluate the space charge characteristics of the samples.

At 30 °C, the space charge property of each material was polarized for 1800 s under an electric field of 40 kV/mm and depolarized for 1800 s by short circuit as shown in Figure 5.

At 30 °C, both B and BP exhibited benign space charge characteristics. There was only a small amount of positive charge injected by the anode in the samples, and the maximum accumulated charge density inside B and BP at the moment of short circuit was 4.9 C/m^3^ and 4 C/m^3^, respectively. The space charge density inside BP with a large amount of vinyl phase removed was lower than that of B. The intensity of the electric field was only slightly distorted, the distortion of the electric field in BP was slightly less than that in B as well.

Different degrees of heteropolar charge generation appeared in both L and LP, and the maximum density was 5.5 C/m^3^ and 5.2 C/m^3^, respectively. At the initial stage of polarization, no heteropolar charges were observed near the anode in L, while stray charges with a maximum density of 1.8 C/m^3^ appeared in the part near the cathode in LP. This phenomenon could be attributed to the difference in the migration rate of positive and negative ions from the impurity dissociation and the distribution of positive and negative charge traps in the samples. The distortion of the electric field at the junction of positive and negative charge packets was relatively serious. The local maximum electric field intensity in L was 54 kV/mm, compared to 41 kV/mm in LP, which is much lower than that in L.

During the depolarization process, all samples showed favorable charge dissipation properties; the residual charge density in all four samples dropped to less than 1 C/m^3^ after 600 s.

When the test temperature rose to 50 °C, the charge injection phenomenon in all samples increased to different degrees. With the passage of time, the charge pack migrated to the interior of the sample, and the charge injection depth gradually increased. The internal charge distribution of the sample during depolarization was more dispersed than that at 30 °C, as shown in Figure 7.

In B, with the extension of polarization time, the accumulation of both positive charge near the anode and negative charge near the cathode of the sample increased with time, while the induced charge on the plate also increased. The maximum accumulated charge density inside the sample at the moment of short circuit was 9 C/m^3^. The charge density decayed with the extension of depolarization and became stable at about 1500 s. At the end of the short circuit, a large amount of positive charge remained near the anode, and the maximum residual charge density inside the sample was about 7.5 C/m^3^. During the short circuit, the accumulated charge inside the sample was homopolar charge. Therefore, it could be inferred that the charge released by the short-circuit process comes from the electrode injection, that is, the Schottky electron emission phenomenon.

Compared with B, with the extension of polarization time, the negative charge density injected by the cathode in BP gradually increased, reaching a maximum of 6 C/m^3^, and the injection phenomenon near the anode was not obvious. At the initial stage of the short circuit, the accumulated charge inside the sample quickly dissipated, becoming stable at about 450 s. At the end of the short circuit, the maximum residual charge density inside the sample was only about 1.5 C/m^3^. According to the electric field distortion, no obvious electricity occurred in both B and BP during the polarization of 1800 s, and the local maximum electric field intensity was 59 kV/mm and 54 kV/mm, respectively. In general, the removal of the vinyl phase does not significantly improve the space charge characteristic of B at 50 °C.

In L, at the initial stage of polarization, a great deal of heteropolar charge was generated near the cathode, and the density of about 11 C/m^3^ was stable during the whole polarization process. A small quantity of negative charge, possibly induced, accumulated inside the sample with a density of about 4.5 C/m^3^. The density of positive charge injected by the anode also increased with polarization time. The maximum accumulated charge density inside the sample at the moment of short circuit was 16 C/m^3^. The maximum local electric field inside the sample reached 66 kV/mm after polarized 1800 s, which increased by 65% compared with the electric field applied, and the electric field distortion was very serious.

Compared with L, the heteropolar charge injection phenomenon disappeared in LP. The density of homopolar charge injected by the electrodes increased with time at the initial stage of polarization, and became stable at about 300 s. The maximum accumulated charge density at the moment of short circuit also decreased, with a value of about 10 C/m^3^. The distortion of the electric field was also improved, and the local maximum electric field was reduced to 53 kV/mm, and the electric field distribution inside the sample was more uniform.

With the further increase of the test temperature, the charge injection phenomenon is further aggravated at 80 °C. A large amount of negative charge injected by the cathode occurred in B at the initial stage of polarization, and the charge density increased with time, reaching a maximum of 12 C/m^3^ at the moment of short circuit. A large amount of heteropolar charge appeared near the anode before 240 s; these heteropolar charges were neutralized by homopolar charges injected by the electrode during the subsequent polarization process. Under the action of the electric field, the positive charge packet migrated to the cathode with the speed decreasing gradually, and stabilized near the negative charge packet near the cathode. The electric field distortion at the junction of the positive and negative charge packets is particularly serious, with a maximum local electric field intensity of 54 kV/mm. At the end of the short circuit, the maximum residual charge density inside the sample was about 2.5 C/m^3^.

In contrast, BP showed a conspicuous inhibition effect of space charge at 80 °C. The phenomenon of charge packet migration did not appear in the sample as in B, and there was no large amount of electrode injection inside the sample either. The maximum accumulated charge density inside the sample at the moment of short circuit was 6.8 C/m^3^. The charge density became stable at about 1500 s after the depolarization began, and the maximum residual charge density was about 5 C/m^3^. Compared with B, the abnormal condition of the electric field had been improved significantly in BP, and the maximum local electric field intensity was reduced to 48 kV/mm. The distortion rate was reduced from 35% to 20%.

At 80 °C, a large amount of heteropolar charge accumulated inside L, and the maximum density of negative charge near the anode reached 15 C/m^3^; a modicum of stray charge could also be observed in the middle of the sample. The distortion of the electric field was most serious at the junction of positive and negative charges, with a maximum of 68 kV/mm, which increased by 70% compared with the electric field applied.

Compared with L, the heteropolar charge injection phenomenon in LP was significantly weakened, and the maximum density of heteropolar charge was about 9 C/m^3^. The negative charge density near the anode was much lower than that in L, and the maximum density of the internal stray charge was only 49% of that in L. The electric field distortion in LP was also well suppressed, with the maximum local electric field at 54 kV/mm. The distortion rate was only about 50% of that in L.

According to the experimental results above, it can be seen that the higher the temperature, the more obvious the electrode injection and dissociation of impurities, and the more space charge accumulated in the material during the polarization. The charge dissipated more slowly during depolarization, and took much longer to stabilize. Both kinds of propenyl samples showed better space charge characteristics than IPC at 30 ° C, 50 ° C and 80 ° C. It could be illustrated that the propenyl samples with most of the vinyl phase removed have fewer interfacial structures with mismatched DC conductivity, according to the current continuity equation, and under the application of an applied electric field, less space charge accumulates inside the material.

The removal of the vinyl phase increases the crystallinity of PP material as a whole, reduces the amorphous region and the two-phase interface, and the charge transfer is much slower in the propenyl phases with less amorphous region and two-phase interface, resulting in the propenyl phases having better space charge properties than the untreated PP material.

At high temperature, the vinyl phase melts into an amorphous state, which cannot effectively block charge transport. Secondly, the temperature increasing speeds up the electron movement, the charge injection and withdrawal rate increase, and the number of carriers and mobility increase, while the injection threshold field strength decreases. The combined effect of the factors above leads to the worse space charge characteristics of PP with a larger amount of vinyl phase than that of the propenyl samples with most of the vinyl phase removed.

## 4. Conclusions

In this work, the vinyl phase and propenyl phase of IPC were separated and the DC conductivity was measured, respectively. The space charge characteristics of untreated IPC materials and propenyl samples with most of the vinyl phase removed were tested. The effects of the mismatch of DC electrical characteristics between different phases on the conductivity and space charge characteristics of impact polypropylene copolymers were studied. The results are as follows.

(1) The DC conductivity of the vinyl phase in IPC is significantly higher than that of the propenyl phase, and the DC conductivity of the two phases presents the characteristics of space charge limited current with the variation of the electric field intensity. In the vinyl phase, due to its unique molecular structure and the flexibility of the chain, charge carriers are easier to migrate under the action of the external electric field, resulting in relatively high DC conductivity. In the propenyl phase, the more rigid molecular structure and crystalline structure hinder the migration of carriers, resulting in lower DC conductivity.
(2) The inhibition effect of propenyl samples with most of the vinyl phase removed on the space charge was enhanced, and the mismatch of the conductivity between the two phases was an important factor affecting the space charge characteristics in the impact polypropylene copolymers. When the vinyl phase was removed, the interfacial structure of the material was reduced, and the interfacial charge accumulation due to the mismatch of the conductivity was reduced, forming a better space charge characteristic. Because the application of cable materials must reduce the hardness of the material, the development of polypropylene DC cable materials should focus on the difference in electrical characteristics between the softening phase and the PP body.

## Data Availability

The original contributions presented in this study are included in the article. Further inquiries can be directed to the corresponding author.
